# Dr. Gilby, on Nitrous Acid in Diabetes

**Published:** 1800-09

**Authors:** William Gilby

**Affiliations:** Physician to the General Hospital, near Birmingham


### *To* Dr. BRADLEY.

SIR,

I Have ſent you four hoſpital caſes of Diabetes Mellitus, three of which have been ſucceſsfully treated, and the other in ſome degree relieved, by the adminiſtration of the Nitrous Acid largely diluted. They are copied from a book wherein I make minutes of all the caſes which come under my care. If you think they deſerve a place in your periodical Work, you are at liberty to inſert them.

The urine of Giſſing and Maſon left a good deal of ſaccharine matter after evaporation, which was of a very dark colour, and of the conſiſtence of very thick treacle. I do not recollect, neither can I aſcertain from the minutes, what proportion the quantity of ſaccharine reſiduum bore to the quantity of urine ſubmitted to the experiment. I ſhould have made ſeveral more experiments upon the urine of the different patients if I had not been too much engaged at the time.

Mr. Chavaſſe, of Walſall, a gentleman of eminence in his profeſſion, has juſt ſent me a caſe of Diabetes Mellitus ſucceſsfully treated by himſelf, which I have alſo incloſed, and which I ſhall be obliged to you to make public.

I am, SIR,

Your obedient humble ſervant,

*July* 31, 1800. WILLIAM GILBY,

Phyſician to the General Hoſpital, near Birmingham.

---

### CASE I.

Anthony Giſſing, aged 42, (admitted January 17,) complains of great weakneſs and inceſſant thirſt. Tongue dry and parched, particularly in a morning; ſkin dry, and harſh to the touch; feels great fulneſs of the abdomen, which is hard and viſibly enlarged; makes 14 pints of urine (which is ſweet and of a pale colour) in the 24 hours, although his drink during this period does not exceed 10 pints; pulſe 90; bowels coſtive; appetite not good, which he attributes to his having lived almoſt entirely upon animal food for ſome months paſt; ſays that he has loſt fleſh very much within the laſt half year.

Preſcription, with reſpect to diet, to live more upon animal than vegetable food, and to have, inſtead of malt liquor, two ounces of brandy mixed with water twice a day.

Capiat ſeri aluminoſi ℥iv, ſextâ quâque horâ et pulveris rhei gr. xv. pro re nata ex quovis vehiculo idoneo.

Jan. 23. Says that he feels himſelf weaker ſince he came into the hofpital, and that the urine, with refpect to quantity, tafte, and colour, is the fame; thirft increafed, but has refrained from drinking, as he did not wifh to exceed 10 pints in the 24 hours; bowels ftill in a coftive ftate.

℞. Decoct. hordei (additis fub finem coctionis rad. glycyrrhiz. incis. ℥iij } ℔ ij.

Acidi nitrofi) - - - - - - - - - - ℥j.

Fiat mift. cujus coch. v. vel vi. ter quaterve intra viginti quatuor horas capiat.

Jan. 26. Reports that he has taken 10 pints of liquids, and made 12 pints of urine, fince the morning of yefterday.

Capiat mift. acid. coch. vj. fextâ quâque horâ.

Jan. 28. Drink, 9 ½ pints in 24 hours; urine, 11 pints, which is ftill pale coloured and fweet; feels lefs pain in the abdomen, which, upon examination, is not fo hard, nor fo much fwelled as it was at the time of his admiffion.

Jan. 30. Drink, 9 pints; urine, 10 pints; complains of pain in his ftomach and bowels. The urine is of a better colour, and lefs fweet.

Omittantur aliquot dofes mifturæ acidæ. Capiat tinct. opii camphorat. ʒj. vel ʒjfs. urg. ventric. vel inteftinorum dolore ex infus. flor. cham. poculo.

Feb. 2. Drink, 8 pints; urine, 9 pints; belly much fofter. The pain having gone off, he yefterday took eight fpoons full of the acid mixture for a dofe.

Sumat mift. acid. dofin fextis horis et bibat fubinde infus. Cinchonæ flavæ poculum.

Feb. 7. Drink, 8 pints; urine, 7 pints, which is not fweet; thirft abated, but is yet troublefome; pulfe 80.

Feb. 9. Drink, 7 pints; urine, 8 pints, which has had no faccharine tafte for the laft five days; feels ftronger; fays he thinks he fhall recover, as the urine has loft its fweetnefs.

Adde mifturæ acidi nitrofi ʒfs. capiat uncias quatuor quintâ quâque horâ.

Feb. 14. The report the fame as on the 9th, except that the urine is rather lefs in quantity.

Feb. 18. Drink, 7 pints; urine, 7 pints.

Feb. 23. Drink, 6 pints; urine, 6 ½ ditto.

Feb. 27. Report the fame as on the 23d; bowels ftill coftive, but free from pain.

Capiat pulv. rhei. gr. xv. ex infus. fennæ ℥ij. pro re nata.

March 6. Drink, 5 pints; urine, 6 pints.

Loco fp. vin. gallic. habeat cerevifiæ fortioris ℔ j. quotidie.

Mar. 15. Report as on the 6th.

Augeatur quantitas cerevifiæ ad ℔ ij. dofin mift. acid. fpatio horarum octo femel tantummodo capiat.

Mar.

Mar. 21. Drink and urine the fame as on the 6th; feels himfelf confiderably ftronger, fince he has had a larger allowance of ale. The urine made in the night is fometimes a little fweet.

April 1. Drink, 6 pints; urine, near 7 pints; complains of pain in the ftomach and bowels.

Capiat opii gr. fs. cum dofibus mifturæ fingulis.

April 8. Report refpecting the urine the fame as on the 1ft inftant. Pain continues.

Capiat opii gr. j. nocte maneque cum mift. acidæ unciis quatuor.

April 15. Drink, 6 pints; urine, 6 pints.

Difcharged at his own requeft.

This patient might have gone on better if he had not been guilty of fome irregularities. He obtained leave to go into the town one afternoon, got intoxicated, and experienced the ufual fymptoms of debility afterwards. His thirft increafed; and he had, for a day or two, an increafed difcharge of urine. At the time he left the Hofpital the urine had its proper colour, tafte, and fmell. I wifhed him to remain fome time longer; but as he had a family, and had regained his ftrength, he wifhed to return to his work. He was defired to fend an account of himfelf at the end of a month, and was promifed a further fupply of medicine if the urine fhould at that time exceed 4 pints in the 24 hours. No account has ever been received either by the apothecary or myfelf. He lives many miles from Birmingham, and I am unacquainted with his addrefs.

### CASE II.

Mary Jeffop, aged 43, admitted Feb. 20th, complains of great thirft, and lofs of appetite. Tongue dry, and very much furred, efpecially in a morning; has great heat at her ftomach, and is frequently troubled with bilious eructations. Says that her belly has become fore and enlarged within the laft fix months. Makes betwixt nine and ten pints of urine in the 24 hours, which is fweet, and of a very pale colour. The quantity of liquids taken within this period, fhe reports to be about four pints and a half. Has a conftant pain in her legs and thighs. Pulfe near 100. Stools regular. Has had no menftrual difcharge for fome months paft.

Capiat mift. acid. ut pro Giffing præfcript. coch. iij. vel iv. octavâ quaque horâ.

Feb. 23. Drink, 4 pints; urine, 8 pints, fince the morning of yefterday, when fhe began to take fix fpoons full of the acid mixture for a dofe.

Adde miſturæ acidi nitroſi ʒſs. capt. coch. vi. ſextâ quâque horâ.

Apr. 25. Drink, 4 pints; urine, 7 pints; in the 24 hours.

Apr. 27. Drink, 4 pints; urine, 6 pints.

March 3. Drink, 4 pints; urine, 5 pints. Pulſe 84; pains in the legs and thighs nearly gone; belly leſs ſwelled, and free from ſoreneſs; thirſt abated; urine, made in the day, not ſweet, and of a much better colour.

March 8. Report reſpecting the drink and urine the ſame as on the 3d. Bowels coſtive.

℞. Pulv. rhei. ʒjſs infus. ſennæ ℔ſs. M. cap. coch. iij vel iv pro re natâ.

Mar. 15. Drink, 3½ pints; urine, 5 pints, which is ſeldom ſweet and of a proper colour. Has had ſeveral purging ſtools in the night. Omittatur miſt. acida.

℞. Miſt. cretacea ℥xiv. ſp. cinamom. ℥ij. M. cap. coch. vi ter quaterve die donec alvus comprimatur. Capiat horâ decubitus opii gr. ſs.

Mar. 21. Drink, 4 pints; urine, 4½ pints. Bowels eaſy, ſtools regular.

Mar. 27. Drink, 3½ pints; urine, 4 pints, which has no ſweet taſte, and is of a proper colour. Says ſhe feels very comfortable, and wiſhes to be diſcharged.

Jeſſop was ſo much reduced by the diſeaſe, that it was quite a fatigue to her to go up ſtairs, or even move about the houſe; to uſe her own words, "She had no power to do any thing." She had been under my care ſeveral months before ſhe came into the hoſpital, had lived as much as poſſible upon animal food, abſtained from all fermented liquors, and had taken moſt of the remedies uſually given in a Diabetes, but without deriving any benefit. At the time of her admiſſion, I directed her to take the common diet of the houſe, but to abſtain from malt liquor; as a ſubſtitute for which, ſhe was allowed to have, now and then, a little very weak brandy and water. This ſoon became diſagreeable to her, and orders were given that ſhe ſhould have half a pint of ale two or three times a day. After ſhe had been in the houſe about ten days, her appetite was much improved, digeſtion tolerably good, and ſhe was able to walk about the ward with very little fatigue.

### CASE III.

John Nichols, aged 45, admitted an out-patient on May 2; complains of great thirſt, ſoreneſs, and fulneſs of the abdomen; makes ten pints of urine in the 24 hours, which is ſweet, and of a very pale colour, but is uncertain as to the quantity of liquids quids which he takes during this period; thinks it does not exceed five pints. Pulſe 86. Says he takes a great deal of food without being ſatisfied.

℞. Acidi nitroſi ʒjſs. decoct. hordei ℔jſs. ſyr. ſimp. ℥ij ft. Miſtura cujus coch. iv. cum aquæ eadem quantitate ter die capiat.

May 13. Liquids drank, 4 pints; urine made, 6 pints. Says he has no ſoreneſs in his belly, very little thirſt compared with what he had ten or twelve days ago. Urine of a better colour, and leſs ſweet.

May 20. Report nearly the ſame as on the 13th; feels much ſtronger; thinks he is able to work.

Capiat miſt. acid. ut antea et bibat decoct. cinchonæ flavæ uncias tres bis die.

May 27. Liquids taken, 3 ½ pints; urine, not 6 pints. Abdomen ſoft, and free from ſoreneſs; thirſt abated; pulſe under 80.

June 3. Liquids taken, near 4 pints; urine 5 pints, which is of a proper colour, and never ſweet. The fulneſs of the abdomen is ſo far gone, that the waiſtcoat which he wore at the time of his admiſſion, and which was then very tight, is now much too wide.

June 10. Liquids drank, near 4 pints; urine, 5 pints. Says he feels nearly well, but not ſtrong enough to go through a day's work.

Omittantur miſt. acid. et decoct. cinchonæ.

℞. Infus. gentianæ comp. ℔j. Acidi vitriolici diluti ʒij. M. cap. coch. iv ter die.

July 13. Called upon me this morning, and ſays that his health was never better. He cannot ſpeak as to the quantity of urine he makes, but knows that it does not exceed what it ought to be.

Nichols never made any alteration in his diet, and never drank any other thing than ſmall beer and cider.

### CASE IV.

John Maſon, aged 23, (admitted January 24) complains of great weakneſs; ſays he has a moſt voracious appetite, and a continual thirſt; makes eleven pints of very ſweet, pale-coloured urine in the twenty-four hours, but cannot ſay what quantity of liquids he takes during this period. Appears much emaciated; pulſe near 100; bowels coſtive.

Capiat miſt. acid. ut pro Giſſing præſcriptæ coch. iv ſextis horis.

Jan. 28. Liquids drank, 7 pints; urine made, 11 pints, ſince

ſince the morning of yeſterday, which is ſtill pale coloured and ſweet.

Capiat infus. ſennæ uncias tres quatuorve quoties alvus ſit aſtricta.

Feb. 4. Report with reſpect to the urine, &c. the ſame as on the 28th ult.

Feb. 9. Liquids taken, 7 pints; urine made, 10 pints. Has pains in his ſtomach and bowels.

Capiat opii pur. gr. ſs. urg. dolore.

Feb. 18. Report the ſame as on the 9th, except that he feels little or no pain. Urine ſweet, and of a pale colour.

Capiat miſt. acid. uncias quatuor quintâ quâque horâ.

Feb. 20. Report as on the 18th, except that the urine made in the day has a ſalt taſte, and is of a higher or deeper colour.

Adde miſt. acid. nitroſi ʒſs capiat doſin eadem quæ 18vo. erat præſcripta.

Feb. 23. Liquids drank, 7 pints; urine, 9 ½ pints, which is of a much darker colour, and ſeldom ſweet. Legs œdematous in the evening.

Feb. 27. Liquids drank, 7 pints; urine, 9 pints.

March 6. Report as on the 27th ult.

Mar. 11. Liquids drank, 7 pints; urine, 8 ½ pints. Complains of great weakneſs. Appetite bad; pulſe 86.

Habeat ceveviſiæ fortioris ℔ſs ter die.

Mar. 18. Liquids drank, 6 ½ pints; urine, 9 pints, which is ſometimes ſweet and at others ſalt. Says that his nights are bad, owing to griping pains, occaſioned, he thinks, by the acid mixture.

Capiat h. s. opii gr. j vel gr. jſs.

Mar. 25. Liquids taken, 6 ½ pints; urine, 8 ½ pints.

April 1. Liquids taken, 7 pints; urine, 9 ½ pints.

Ap. 8. Report as on the 1ſt. Wiſhes to give over taking medicines, and to try whether a month's reſidence in the country will be of any ſervice, as he begins to loathe all kinds of food.

Omittatur miſt. acid. Bibat aquæ calcis uncias quatuor quartis horis.

Ap. 15. Liquids taken, 7 pints; urine, 9 pints.

Diſcharged.

May 13. Has been drinking the lime-water ever ſince the 15th of laſt month; and ſays that his urine is of a pale colour, and ſweet; and that he generally makes about ten pints in the 24 hours. Appetite is more keen ſince he left the hoſpital.

The acid medicine has not done much for this patient. He lived for ſome weeks almoſt entirely upon animal food, and then then faid it went fo much againft his ftomach that he could not get down any more, unlefs he were to have an allowance of bread and malt liquor.

