# Errata

**Published:** 1800-10

**Authors:** 


### ERRATA.

Vol. IV. p. 208, l. 16, for *cretacea*, read *cretaceæ.*

210, l. 16, for *nitrorsi*, read *nitrosi*; and for *eadem*, read *eandem.*

